# Supplementary material for: Molecular Correlates of Diapause in Aphidoletes aphidimyza
Source: Insects. 2024 Apr 23;15(5):299. doi: 10.3390/insects15050299 (PMC11122395; doi:10.3390/insects15050299)
Supplement: Supplementary file 1 [file insects-15-00299-s001.zip › supplementary/Table S1.pdf]

Supplement table XIII Primer sequences for qRT-PCR

Gene ID	Gene name	Primer sequence
c51531.graph_c2	Tubulin	GGACAATCTGGTGCCGGTAA<br>GCAATCGCATGATTCGGCTT
c58243.graph_c1	Citrate synthase (CS)	GGGTATTCGCTTCCGTGGAT<br>TCACCGGTAACAAGCAACCA
c62192.graph_c1	Isocitrate dehydrogenase (IDH)	AGGCGGTACGGTATTTCGTG<br>ATTGATCAGCGTGGGCATGT
c62223.graph_c12	Oxoglutarate dehydrogenase (OGDH)	GCCCAGTTTCGACTGTACCA<br>TTCAGTTCGTGTGCCGGTAA
c61907.graph_c1	Succinate dehydrogenase (SDH)	TGCGACTGCTTTGTGTGTTG<br>ATGAATGCCAATGTTGCGGC
c61013.graph_c5	Malate dehydrogenase (MDH)	ATTGGTGGTCATTCGGGTGT<br>TGAATGCGTTCGGTCAATGC
c58815.graph_c0	Phosphoglycerate kinase (PGK)	ATACACTTTGGCTCCGGTGG<br>AAGCCTCTTCAACTTCGGCA
c59467.graph_c0	Pyruvate carboxylase (PC)	GTGACGTAAGCGATCCGACT<br>CAAAGCACATGAGTTCCGGC
c62150.graph_c0	Phosphoglucomutase (PGM)	AATTGCCGCTTTGCTTGGTT<br>AACAGTTTCGAGCTCACCGT
c60574.graph_c0	Glucose-6-phosphate 1-dehydrogenase (G6PD)	ATGAACGCTGGGATGGTGTT<br>AAATATCGCCGGGCACATCA
c60958.graph_c0	Glucose-6-phosphatase 2 (G6P)	TCACATGCGAAACAACTGCG<br>CGACGAAACCAAGGCGATTG
c61092.graph_c9	Glycogen phosphorylase (GP)	CCATCCCAGAGCTTATGCGT<br>CCTCCGGTAAAACGGTGTGA
c56307.graph_c0	Alpha-trehalose-phosphate synthase (TPS)	TGGTGTCGATTGGTCTGAGC<br>TGTGATGATGTGACGCGGAA
c60207.graph_c1	Trehalase (TREA)	AAAAGCTGGCGCTGAAAGTG<br>AACCGGAACAATTGAACGGC
c51671.graph_c0	Trehalose-phosphate phosphatase (TPP)	AAAGGTCATGCTGCGCTACT<br>GTCTTCATCGCTCGTGTCGT
c38842.graph_c0	Glycogen synthase (GYS)	TGAACTACGGGACCCAAACG<br>CAGGGATGAGGATCGAAGCC
c41125.graph_c0	Phosphoenolpyruvate carboxykinase (PEPCK)	TGGTGTTGCACCAGGAACAT<br>CAGTAAACACCACCGTCCGA
c54598.graph_c0	Hexokinase（HK）	ATGGGGCGCATTTGGTGATA<br>CCTCCCGGCAAAAGAGATGT
c61807.graph_c1	6-phosphofructokinase	CTTGCGGTATGAATGCAGCC<br>TTTGCTTGACATTGCCAGCC
c59857.graph_c0	Glucose-6-phosphate isomerase	TGCGATTTTATTGCACCGGC<br>AGCTTCGGTTTTACCAGCCA
c54154.graph_c0	Phosphoglycerate mutase	CACCAAGGATCCCCGGTATG<br>CCAGTATGGCAGTGTACGGG
