# Supplementary figures and images for: Molecular Correlates of Diapause in Aphidoletes aphidimyza
Source: Insects. 2024 Apr 23;15(5):299. doi: 10.3390/insects15050299 (PMC11122395; doi:10.3390/insects15050299)

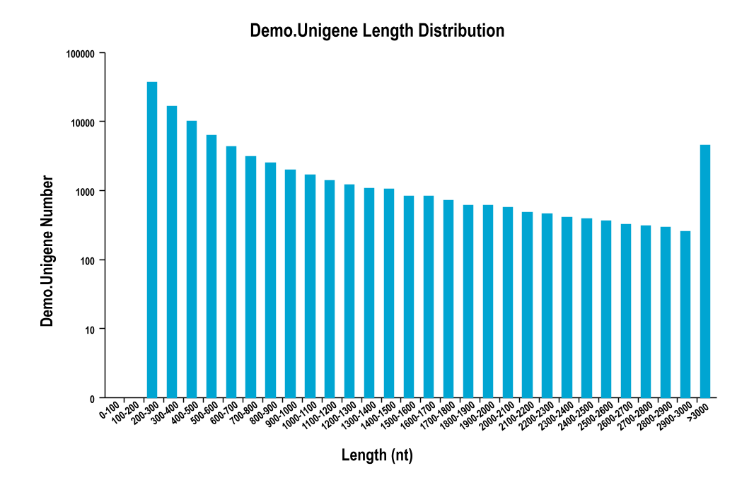

Supplement: Supplementary file 1 [file insects-15-00299-s001.zip › supplementary/Figure S1.png]

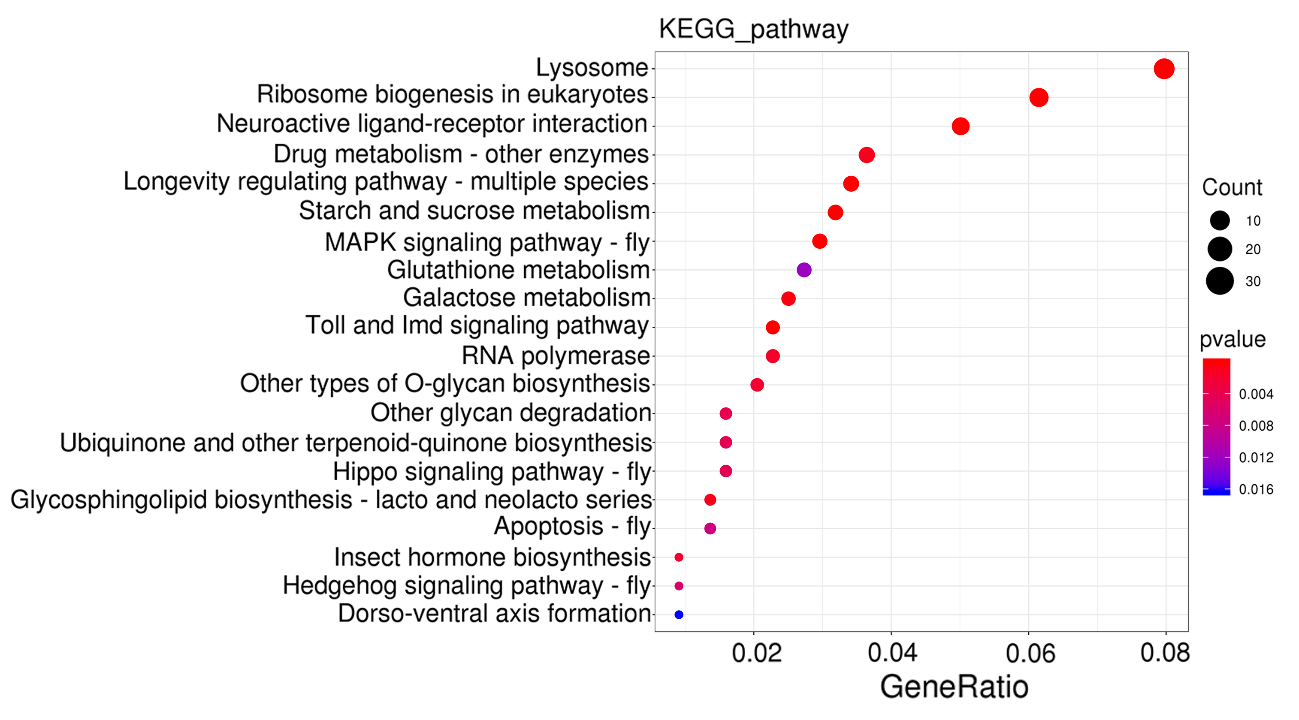

Supplement: Supplementary file 1 [file insects-15-00299-s001.zip › supplementary/Figure S10.png]

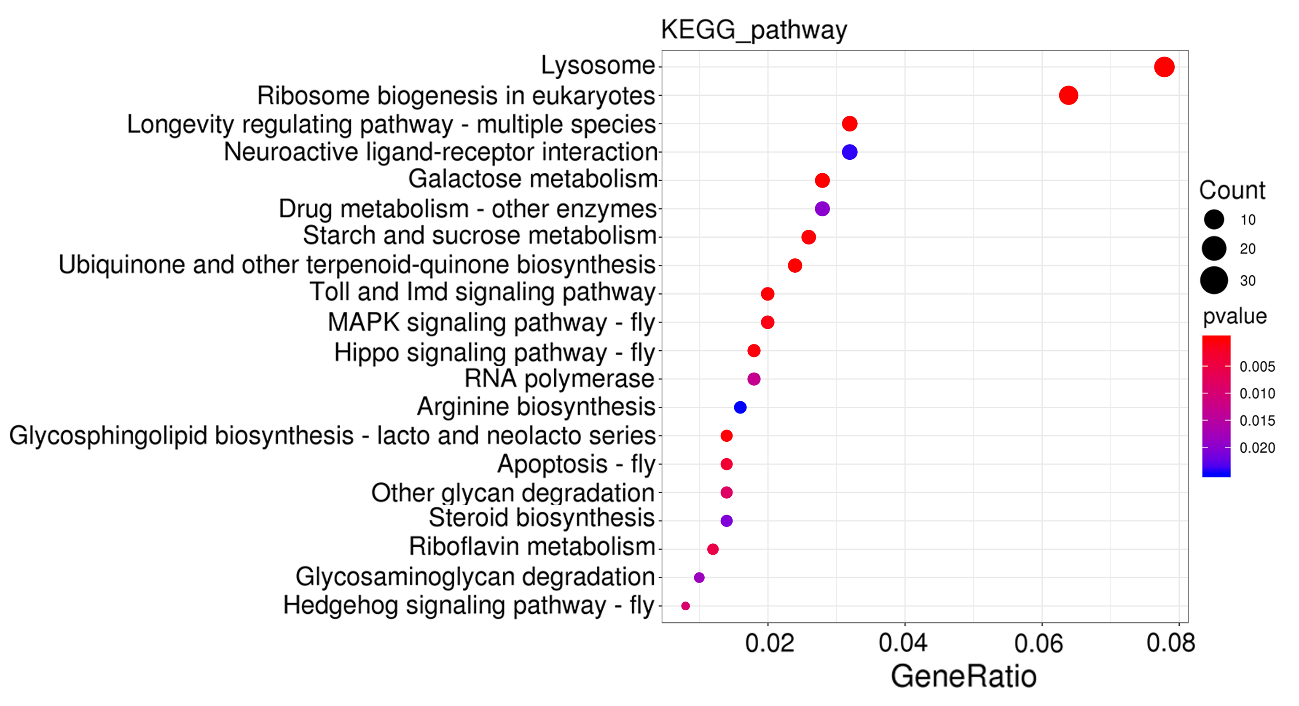

Supplement: Supplementary file 1 [file insects-15-00299-s001.zip › supplementary/Figure S11.png]

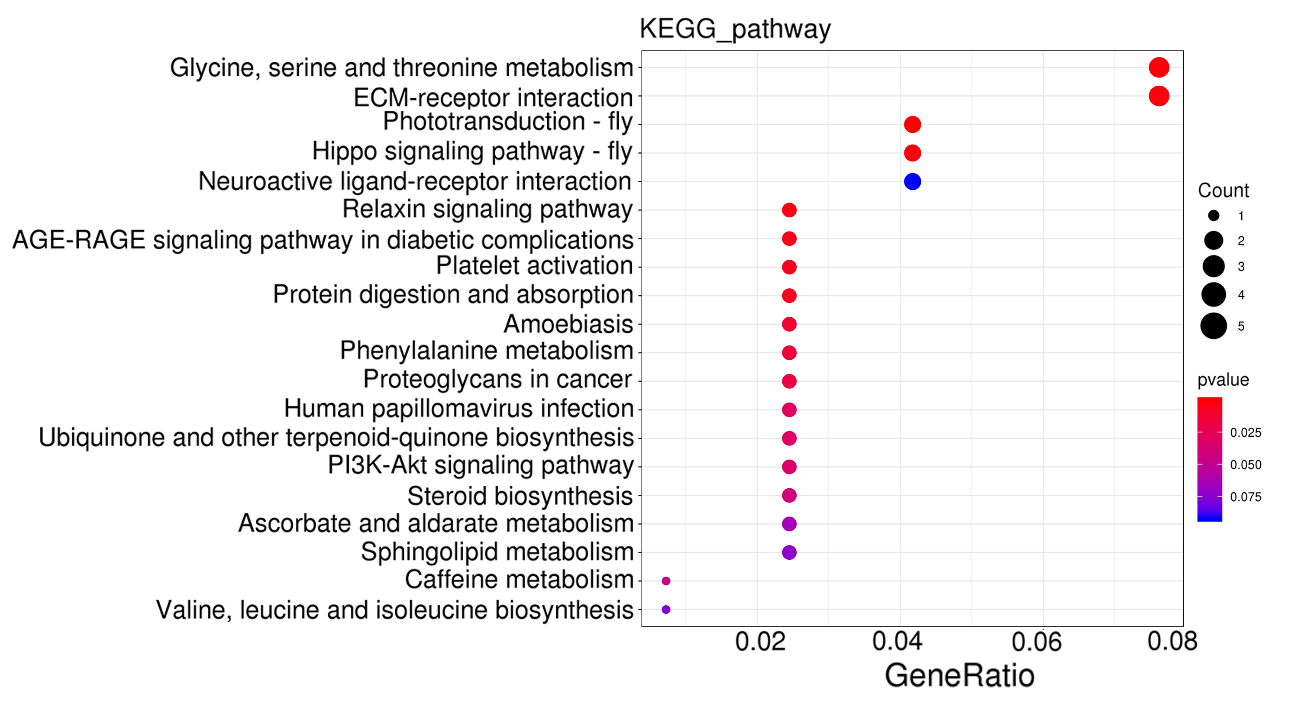

Supplement: Supplementary file 1 [file insects-15-00299-s001.zip › supplementary/Figure S12.png]

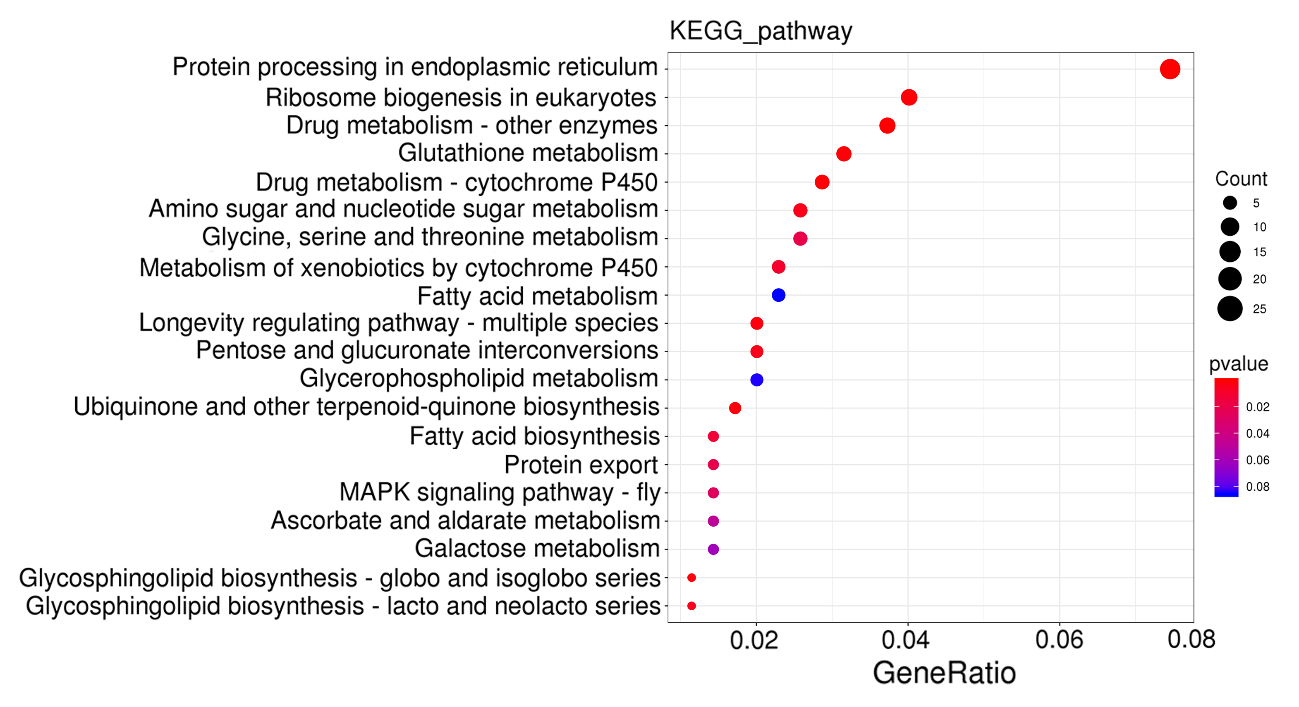

Supplement: Supplementary file 1 [file insects-15-00299-s001.zip › supplementary/Figure S13.png]

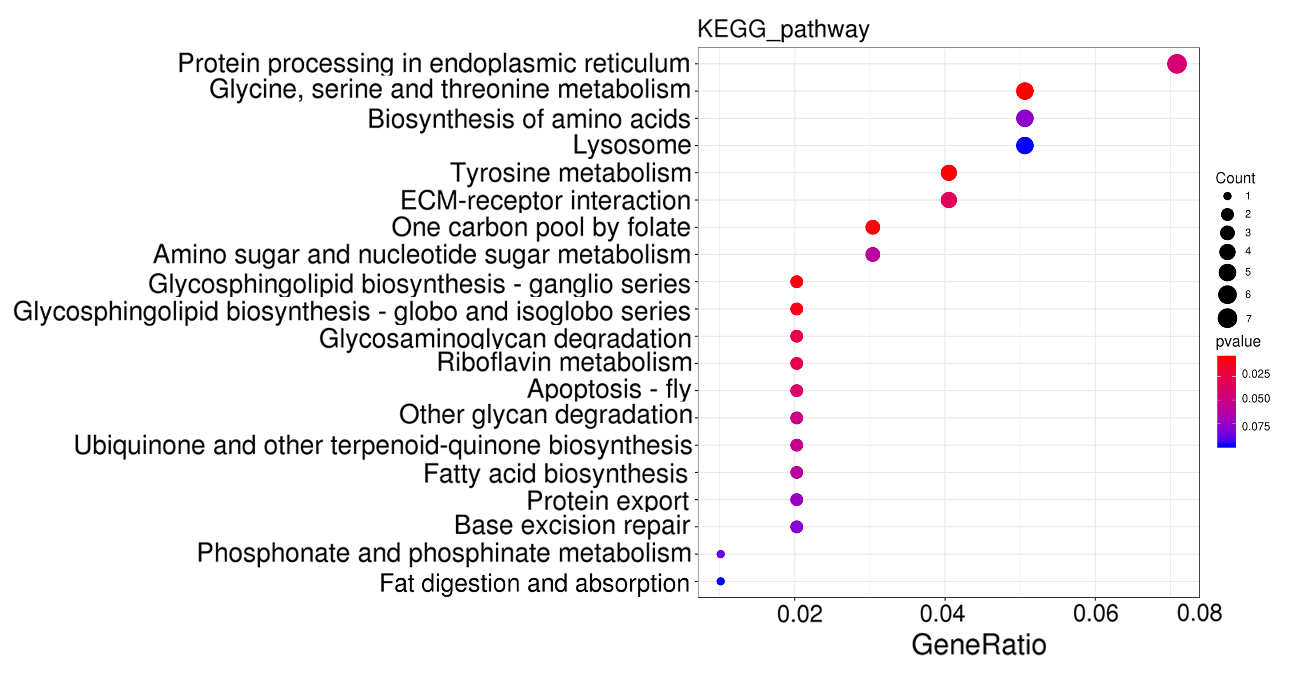

Supplement: Supplementary file 1 [file insects-15-00299-s001.zip › supplementary/Figure S14.png]

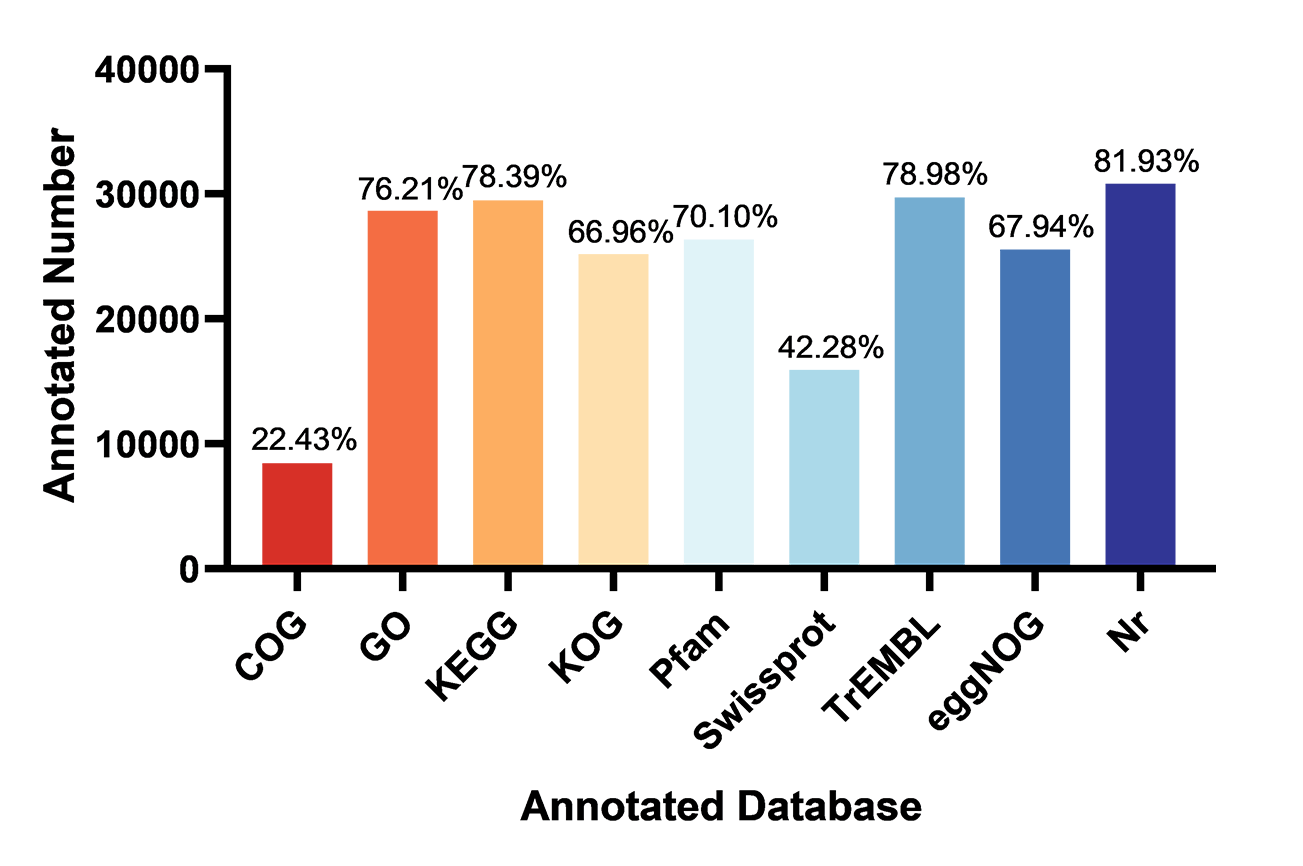

Supplement: Supplementary file 1 [file insects-15-00299-s001.zip › supplementary/Figure S2.png]

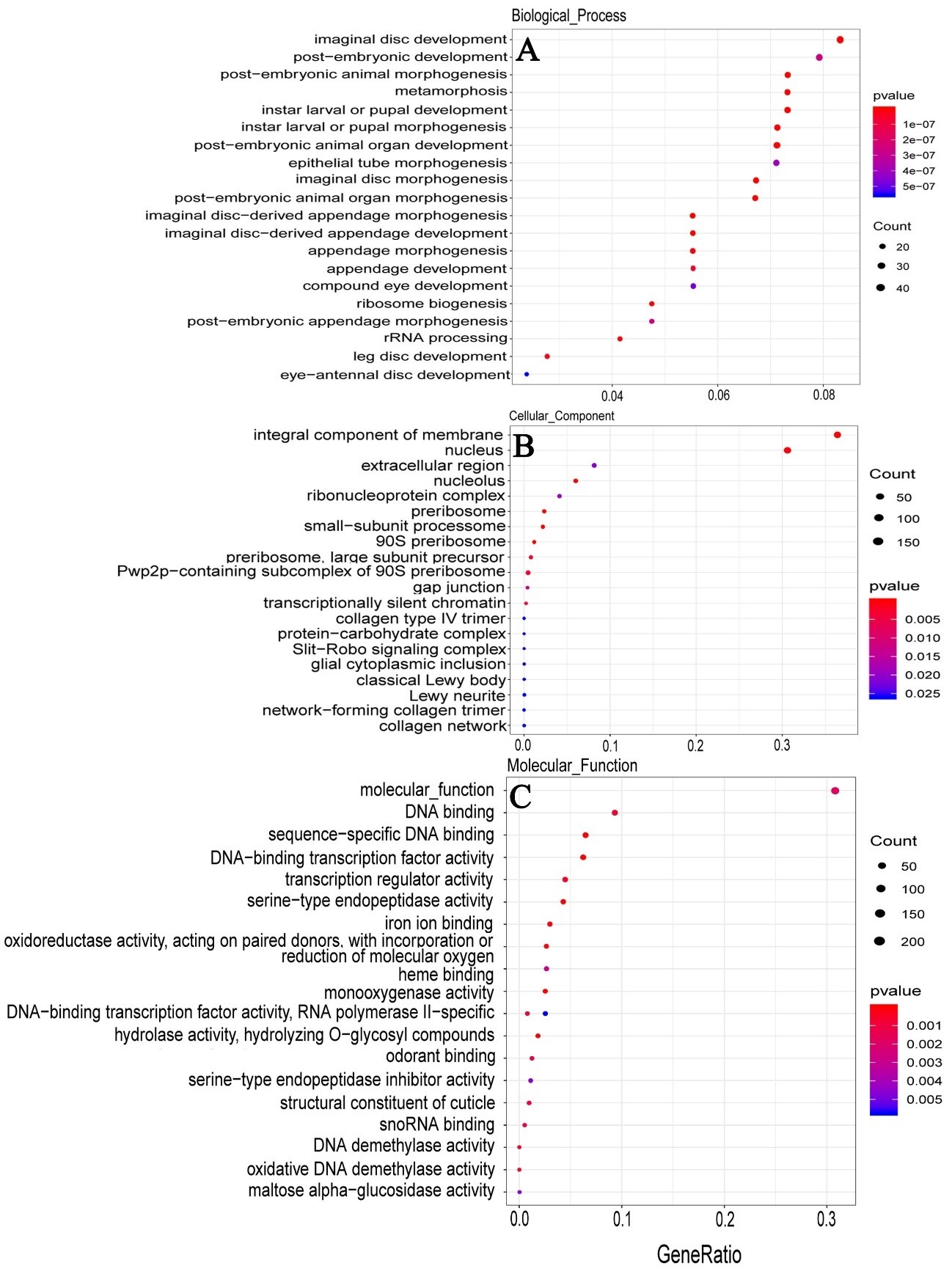

Supplement: Supplementary file 1 [file insects-15-00299-s001.zip › supplementary/Figure S3.jpg]

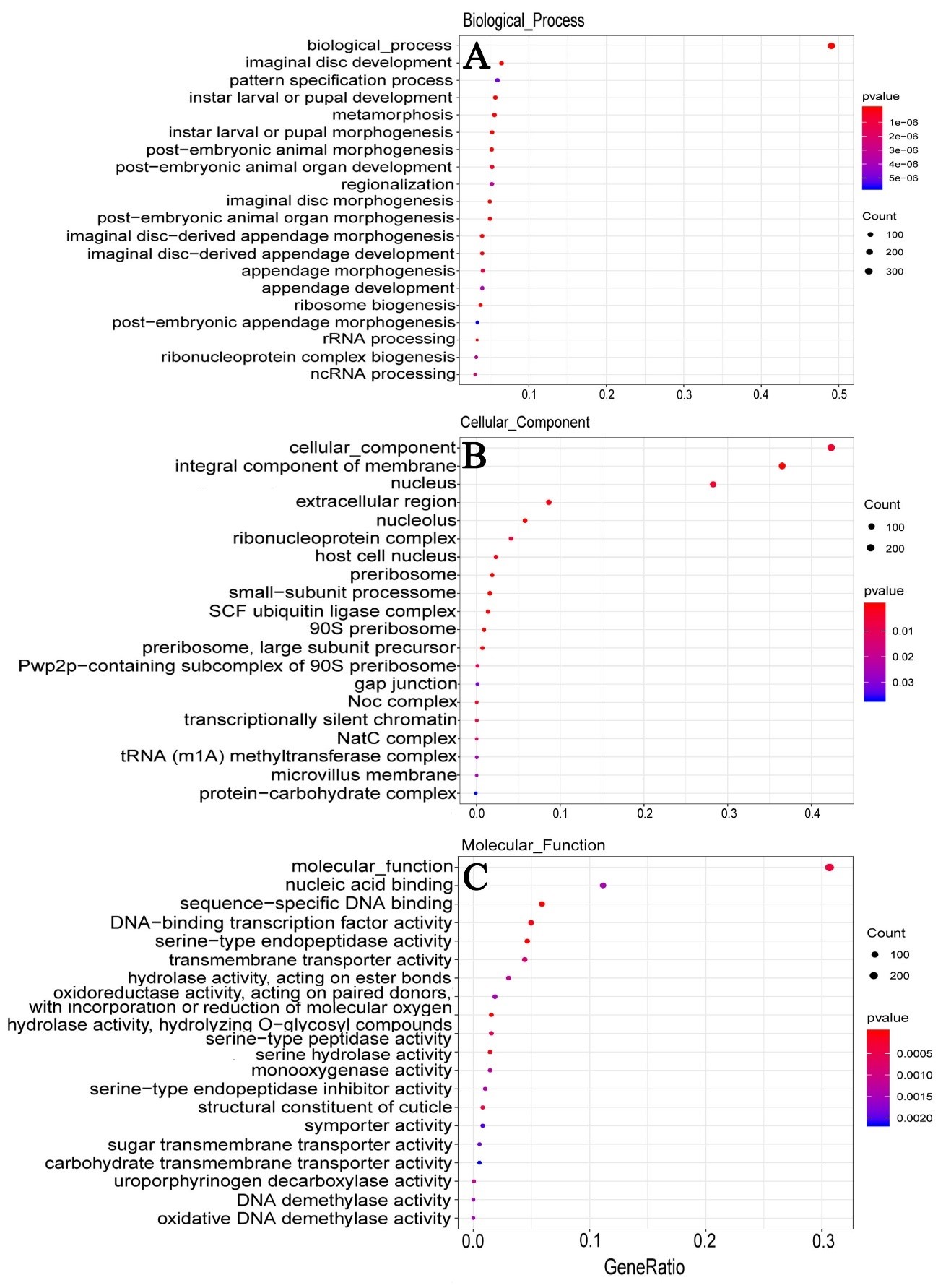

Supplement: Supplementary file 1 [file insects-15-00299-s001.zip › supplementary/Figure S4.jpg]

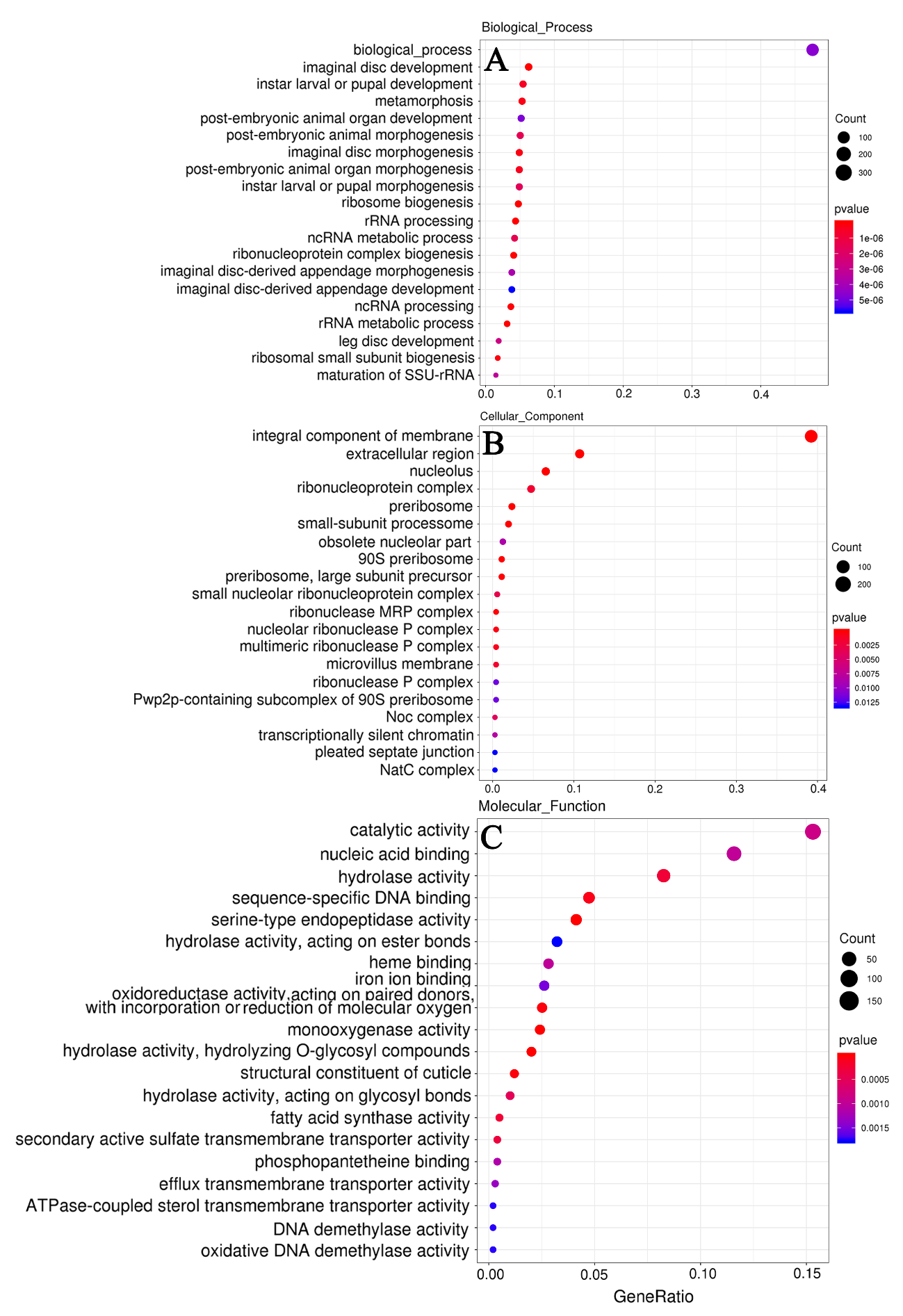

Supplement: Supplementary file 1 [file insects-15-00299-s001.zip › supplementary/Figure S5.png]

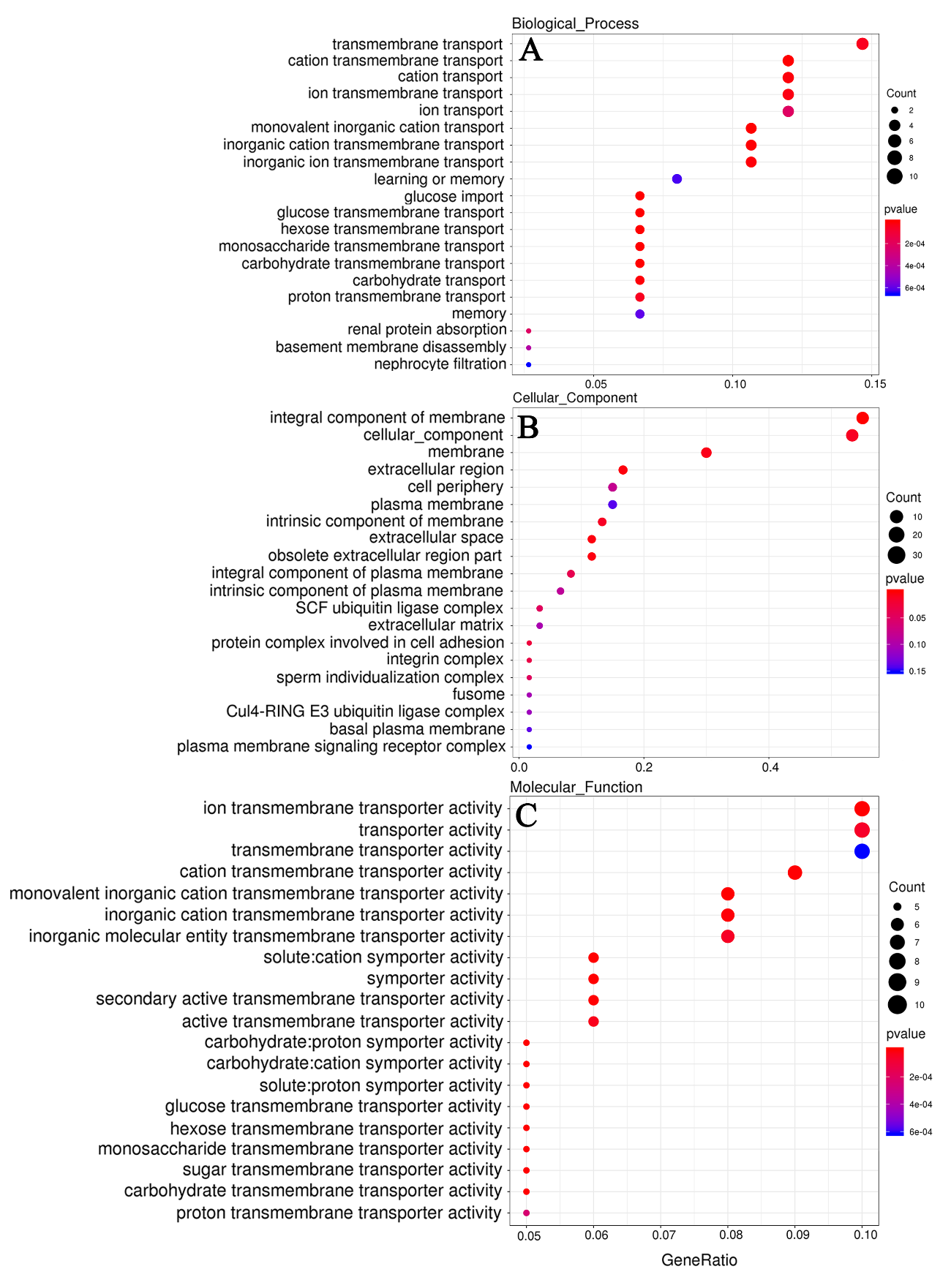

Supplement: Supplementary file 1 [file insects-15-00299-s001.zip › supplementary/Figure S6.png]

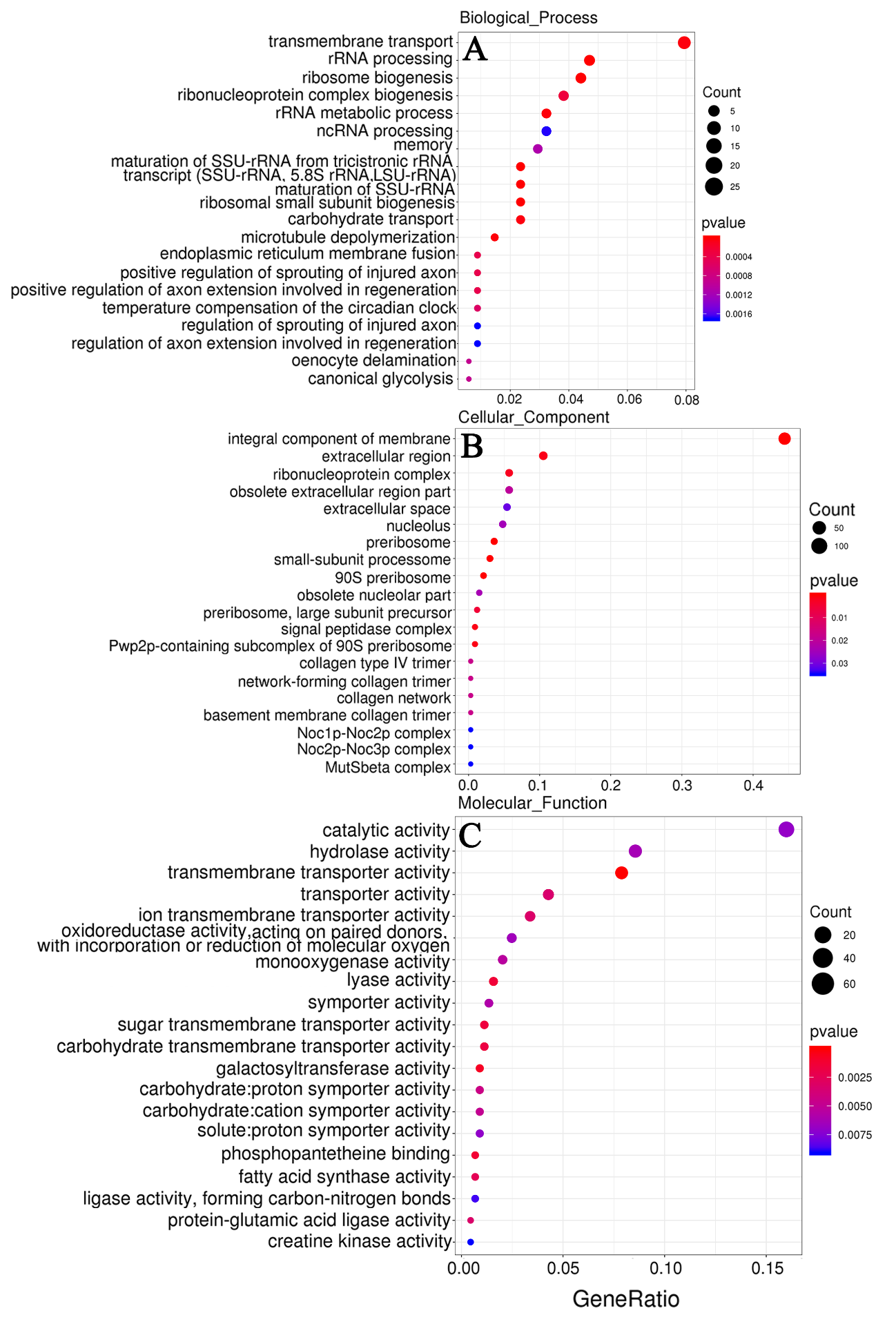

Supplement: Supplementary file 1 [file insects-15-00299-s001.zip › supplementary/Figure S7.png]

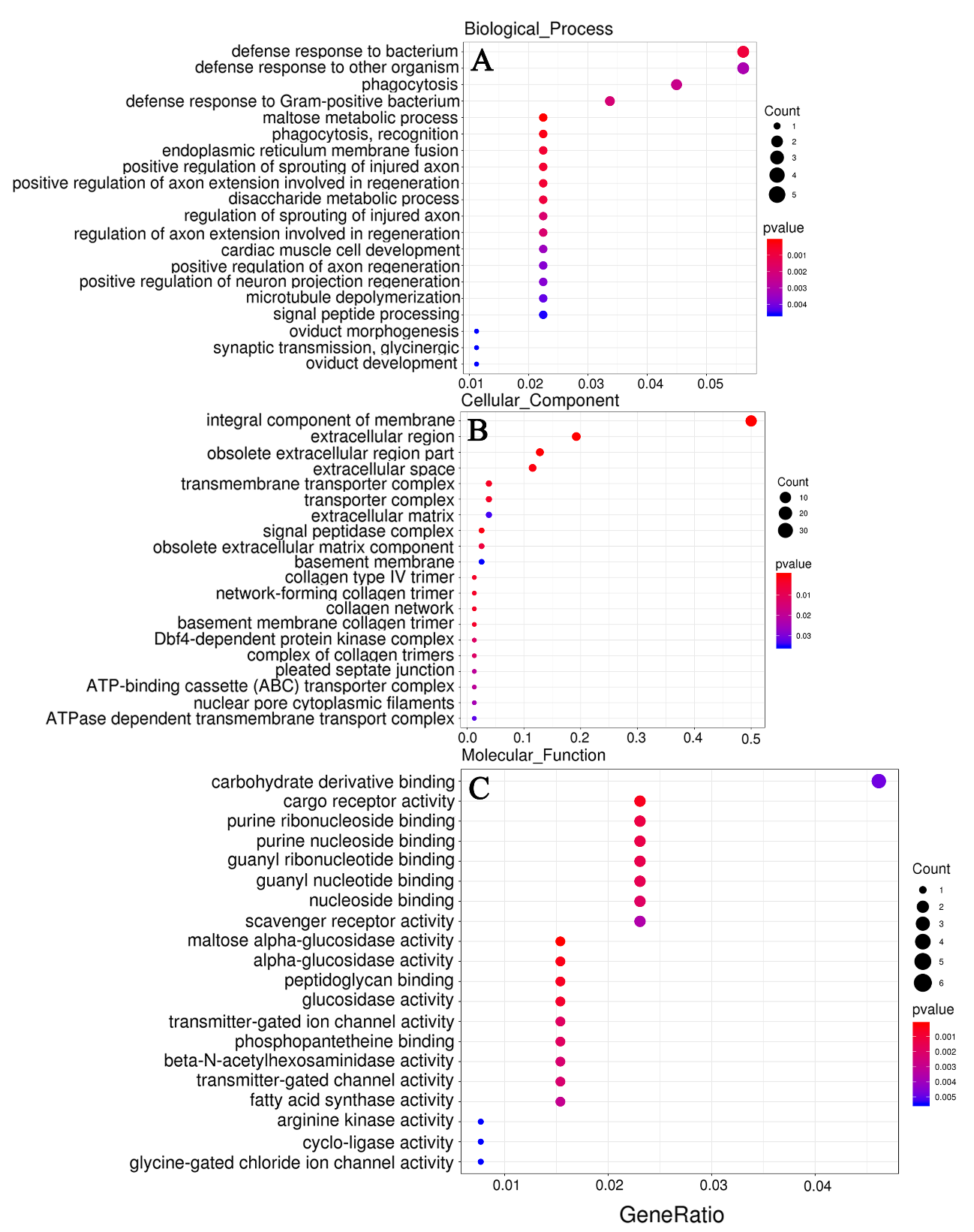

Supplement: Supplementary file 1 [file insects-15-00299-s001.zip › supplementary/Figure S8.png]

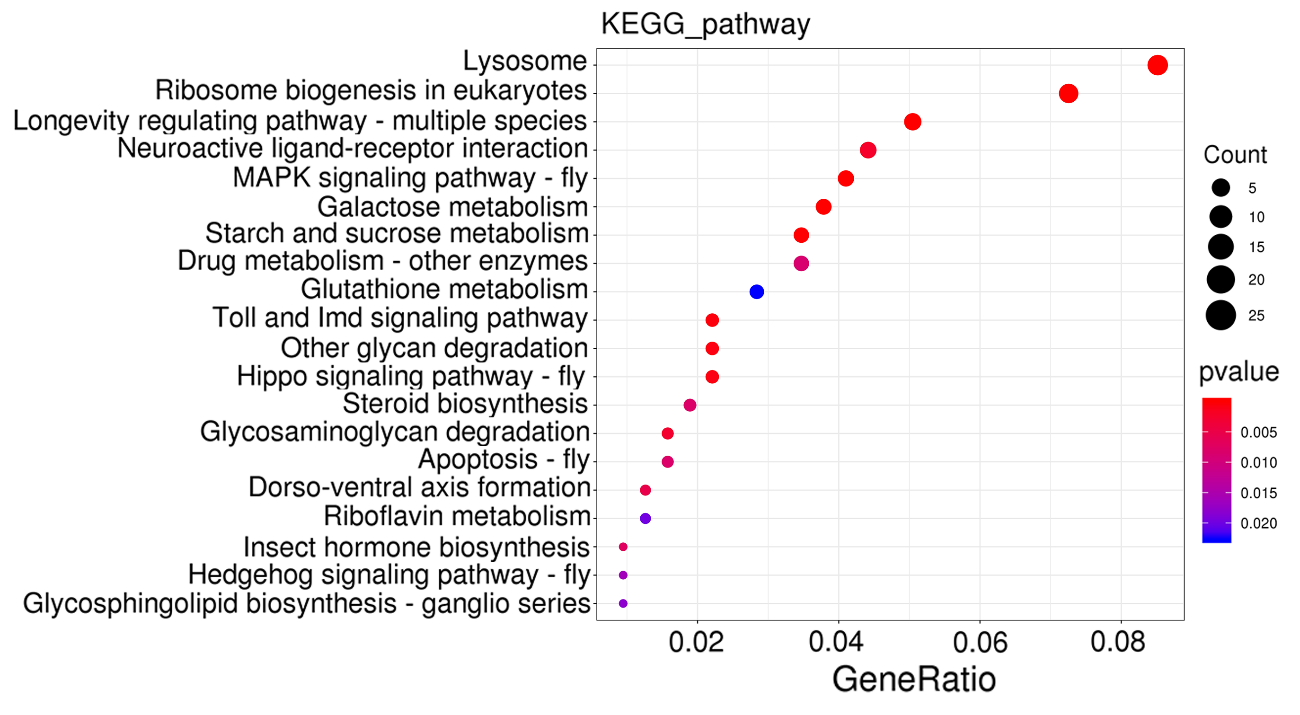

Supplement: Supplementary file 1 [file insects-15-00299-s001.zip › supplementary/Figure S9.png]
